# Trends of Acute Renal Colic During COVID-19 Lockdown: An Experience From Saudi Arabia

**DOI:** 10.7759/cureus.29481

**Published:** 2022-09-23

**Authors:** Yahya Ghazwani, Rakan Aldarrab, Mohammad A Alghafees, Saeed Bin Hamri, Khalid Alrabeeah, Abdullah Alkhayal, Fisal T Aldokhel, Abdulrahman K Alageel, Mohammed K Alageel, Mohammed Q Alosiami, Almohannad K Alqarni, Yasser A Noureldin

**Affiliations:** 1 Urology, King Abdulaziz Medical City Riyadh, Riyadh, SAU; 2 Medicine, King Saud Bin Abdulaziz University for Health Sciences, Riyadh, SAU; 3 Urology, King Abdullah International Medical Research Center, Riyadh, SAU; 4 Medicine, King Saud University, Riyadh, SAU

**Keywords:** urologic emergency, lockdown, endourology, renal colic, covid-19

## Abstract

Introduction

The COVID-19 pandemic represents an unprecedented challenge for healthcare systems around the world. Saudi Arabia was one of the first countries to experience a lockdown and postponement of elective surgical procedures. The objective of this study was to assess the trends of acute renal colic presenting to our emergency department.

Methods

This retrospective study targeted all patients who presented with acute renal colic during the lockdown period (March 23, 2019 to June 20, 2019). Patients’ and stone data were collected. The patient’s data included age, gender, BMI, and comorbidities. Stones' data included stone size, location, side, evidence of obstruction and UTI, and planned and conducted management.

Results

A total of 137 patients were identified; 92 (67.2%) patients were males with a mean age of 44 ± 16 years. Positive history of urolithiasis was reported in 47 (34.3%). The most common initial investigation was non-contrast CTs (93.4%). The majority of patients had a stone size of < 10 mm (93%) and ureteric stones (81.2%). A total of 32 patients (32.4%) had evidence of UTI and 63.4% had evidence of obstruction. Most of the patients (73.7%) were offered medical expulsive therapy (MET). Only 2.2% did not receive the planned management.

Conclusion

The observed pattern shows that the management during the lockdown did not differ from the original recommendations. This could be due to the fact that most patients had stone sizes between 5 and 10 mm and consequently were managed by METs. Larger data need to be conducted to provide concrete evidence. Such data are relevant to provide a clear guide for management and to establish protocols for emergency lockdown situations.

## Introduction

Acute renal colic is defined as a sudden and severe form of pain that results due to the presence of a stone in the urinary system. The stone can occur anywhere along with the urinary tract system. Typically, pain originates over the costovertebral angle and extends anteriorly and inferiorly towards the groin or testicles [1]. Symptoms of renal colic include but are not limited to flank pain, abdominal pain, back pain, nausea and vomiting, fever, and dysuria [2]. The prevalence of urolithiasis in Saudi Arabia was 6.2%, including 6.6% males and 5.8% females [3]. The diagnosis is mainly reached by taking a detailed history, performing a physical exam, and radiological imaging, including Ultrasonography, and non-contrast helical computed tomography [4-6]. Out of these imaging modalities, non-contrast computerised tomography has become the recommended imaging study to establish the diagnosis of renal colic due to its high sensitivity and specificity [5.6]. The treatment includes observation, medical expulsive therapy, pain management, and surgical intervention [7-9]. Stones can lead to many complications: renal failure, ureteral stricture, infection, urine extravasation, and perinephric abscess [10].

COVID-19 is a viral infection caused by the newly discovered coronavirus [11]. In March 2020, the World Health Organization announced that COVID-19 is a pandemic [12]. Therefore, many countries, including Saudi Arabia, took precautionary measures to decrease the spread of the infection, decrease the chance of overloading health facilities, and protect their population from COVID-19 infection, including curfew 24 hours a day partial curfew, and postponement of the elective surgery. These precautionary measures might lead to delay in the presentation of renal colic patients, which can advance the severity of the clinical condition with an increase in the chances of the complication.

To our knowledge, some studies assess the trend of renal colic during the COVID-19 pandemic. A study was conducted in Italy that compared the visits during the pandemic with the previous year. The study showed a decrease in the urgent visit during the pandemic by 38.9%, urgent visits of patients who accessed directly from the emergency department by 51.1%, whereas the number of urgent care visits referred by GP remained unchanged. Consequently, the rate of visits from ED decreased from 75% to 60%, and the rate of visits requested by GP increased from 25% to 40%. Notably, the rate of visits for renal colic, LUTS, and other not precisely defined disorders from ED decreased, and the corresponding rates of visits of patients referred by GPs increased [13]. Moreover, there is a study conducted in Saudi Arabia that includes multicenter. They compare the COVID-19 era versus the same months in 2019. The study showed a decrease in elective procedures by 34.3% in the 1st third of 2020 compared to the first third of 2019. At the same time, emergency procedures showed a 9.3% decrease between the aforementioned time frames [14]. We aimed in this study to assess the trend of acute renal colic and their management during the COVID-19 lockdown period.

## Materials and methods

This retrospective cross-sectional study was conducted at King Abdulaziz Medical City (KAMC) in Riyadh, Saudi Arabia. KAMC is a tertiary medical center with a 1501-bed capacity. This study was designed to assess the trend of renal colic presentations, management, and outcomes during the COVID-19 lockdown period.

We included all patients who presented to the emergency department at KAMC with renal colic during the lockdown period (March 23, 2019 to June 20, 2019). The electronic medical records of patients who met the included criteria were reviewed. We used customized data collection sheet that includes baseline characteristics, comorbidities, initial investigation, stone feature, management, and outcome. 

The data were analysed by using SPSS v26 (IBM Corp., Armonk, NY). The descriptive statistic will be presented as frequency and percentage for the categorical data variables and mean and standard deviation for the numerical data. The study was approved by the Institutional Review Board (IRB) committee (NRC21R/276/06) at King Abdullah International Medical Research Center (KAIMRC), the Ministry of National Guard Health Affairs.

## Results

In total, 137 patients were identified during the lockdown period; their demographics are presented in Table 1. Ninety-two patients (67.2%) were males with a mean age of 44 ± 16 years. The mean body mass index was 28.8 ± 7.4 kg/m2. Out of the total patients, 48 patients (35%) suffered from one or more chronic diseases. The most common chronic disease was diabetes mellitus, followed by hypertension, dyslipidaemia, and hyperuricemia with a prevalence of 24.1%, 23%, 16.1%, and 0.7%, respectively.

**Table 1:** Baseline demographic characteristic and comorbidities of patients (N = 137)

Characteristics		
Age: Mean ± SD		44 ± 16 years
Gender: N (%)	Male	92 (67.2%)
	Female	45 (32.8%)
BMI: Mean ± SD		28.8 ± 7.4 kg/m^2^
Prevalence of comorbidities: N (%)		
	Diabetes mellitus	33 (24.1%)
	Hypertension	23 (23.4%)
	Dyslipidemia	22 (16.1%)
	Hyperuricemia	1 (0.7%)

For the diagnosis assessment and presentation of the patients, as shown in Table 2, out of 137 patients, 47 (34.3%) of them had a history of renal stones. The most common initial investigation ordered were non-contrast CTs, ordered for 128 of the patients (93.4%), followed by ultrasound for five patients (3.6%), followed by x-ray for one patient. However, three patients (2.2%) were diagnosed without any imaging studies. The mean size of stones was 5.2 ± 4 mm. The majority of the patients (86.9%) had a stone size of less than 10 mm. Regarding the stone location, the majority (78.8%) had ureteric stones. Most of the patients (43.1%) were on the left side. Moreover, 85 patients (62%) had evidence of obstruction. Among our patients, 32 patients (32.4%) had evidence of a urinary tract infection.

**Table 2:** Patients’ presentations and diagnosis approaches

Parameter		
History of previous stones: N (%)	Yes	47 (34.3%)
	No	90 (65.7%)
Initial investigations: N (%)	Not-contrast CT	128 (93.4%)
	Ultrasound	5 (3.6%)
	X-ray	1 (0.7%)
	No test was ordered	3 (2.2%)
Stone size in mm: Mean ± SD		5.2 ± 4 mm
Stone size classification: N (%)	Less than 10 mm	119 (86.9%)
	More than or equal to 10 mm	9 (6.6%)
	Missing	9 (6.6%)
Stone location: N (%)	Renal	25 (18.2%)
	Ureteric	108 (78.8%)
	Missing	4 (2.9%)
Stone side: N (%)	Right	39 (28.5%)
	Left	59 (43.1%)
	Bilateral	36 (26.3%)
	Missing	3 (2.2%)
Evidence of obstruction: N (%)	Yes	85 (62%)
	No	49 (35.8%)
	missing	3 (2.2%)
Evidence of urinary tract infection: N (%)	Yes	32 (23.4%)
	No	105 (76.6%)

For the management, as shown in Table 3, The majority of the patients (73.7%) were planned to be managed by medical expulsive therapy. Out of 137 patients, only three patients (2.2%) did not receive the suggested management. Regarding analgesic use, 129 patients (94.2%) received analgesic medications. Paracetamol was the most common medication, followed by NSAIDs then opioids by 74.5%, 48.2%, and 36.5%, respectively. Out of 137 patients, only 43 (31.4%) had a follow-up with a mean day of 39.7 ± 48.9 days. Stones passed spontaneously in 15 patients and were removed by ureteroscopy in nine patients.

**Table 3:** Patients’ management and follow-up

Parameter		
Suggested management: N (%)	Medical expulsive therapy	101 (73.7%)
	Direct ureteroscopy	17 (12.4%)
	Stenting	19 (13.9%)
Receiving the suggested management: N (%)	Yes	134 (97.8%)
	No	3 (2.2%)
Receiving analgesic: N (%)	Yes	129 (94.2%)
	No	8 (5.8%)
Types of analgesic: N (%)	Paracetamol	
	Yes	102 (74.5%)
	No	35 (25.5%)
	NSAIDs	
	Yes	66 (48.2%)
	No	71 (51.8%)
	Opioid	
	Yes	50 (36.5%)
	No	87 (63.5%)
Receiving antibiotics: N (%)	Yes	51 (37.2%)
	No	86 (62.8%)
Follow-Up: N (%)	Yes	43 (31.4%)
	No	94 (68.6%)
Mean duration of follow-up: Mean ± SD		37.9 ± 48.9 days
Definitive stone removal: N (%)	Spontaneous passage	15 (10.9%)
	Ureteroscopy removal	9 (6.6%)

## Discussion

The current literature suggests that treatment options for acute renal colic management have notably changed during the national COVID-19 lockdown, with a significant decrease in the number of emergency room visits and hospital admissions being observed [15]. However, our findings collectively contradict several reports in the current literature and demonstrate that the management of patients during the lockdown period did not differ from the original recommendations.

Diagnosis assessment 

Non-contrast CTs were ordered for 93.4% of the cohort, consistent with the current literature. The current literature also suggests that the number of CTs performed during the pandemic was greater than that of pre-COVID. Anderson et al. sought to examine the impact of COVID-19 on acute urolithiasis presentations to the ED, retrospectively reviewing all non-contrast CTs of the kidneys, ureter and bladder. It was noted that, of 198 CT scans, 94 were performed pre-COVID, and 104 were performed during the pandemic. This remained the primary form of diagnosis assessment [16].

Management

All patients in our cohort aside from three received the suggested management approach, primarily analgesic medications (94.2%). Paracetamol represented the most commonly used medication (74.5%), followed by NSAIDs (48.2%) and opioids (36.5%). Surgical intervention was required in nine patients (6.7%). The mean follow-up period was 39.7 days. Similar management was implemented across 14 patients infected with COVID-19 who presented with acute renal colic. Demirdogen et al. reported that analgesic treatment was adopted in 78.6% of patients, with complete pain control being achieved in 64.2% of patients. However, surgical intervention was required in 35.7% of patients, significantly higher than that of our cohort [17].

These findings were concurrent with that of Carrion et al. that hypothesised the COVID-19 pandemic and subsequent national lockdown would delay renal colic patients presenting to the Emergency Department (ED), resulting in more severe clinical conditions at presentation. However, this retrospective review noted that, although a significant reduction in ED presentations for renal colic was observed in both Spain and Italy, there were no significant differences observed concerning the length of stay, Urology department admission requirement and type of therapy administered [18]. Anderson et al., on the other hand, contradicted this, emphasising that the COVID-19 pandemic and subsequent national lockdown did not result in fewer patients presenting with acute ureteric colic cases to the ED [16].

Yasseri et al. suggest that a preventative approach be taken for patients for whom the urological intervention is not indicated. This involves general dietary and lifestyle recommendations and was more widely adopted in the current literature during the COVID-19 pandemic. On the other hand, in patients where urological intervention is required, patients can be deemed emergent or nonemergent. Emergent cases often involve surgical intervention, with the preferred option of percutaneous nephrostomy tube insertion under local anaesthesia. In nonemergent cases, conservative treatment is recommended for three months during the COVID-19 lockdown, after which time a re-evaluation can be conducted [19,20]. These recommendations are reflected in our cohort; however, the mean follow-up was substantially lower amongst our cohort than the recommended three months.

Limitations

There were a few limitations to the presented study. Firstly, the data registered in the electronic health record system may have been incomplete. Secondly, the research team did not directly observe all of the cases; as such, they had to rely on others for accurate recordkeeping. For example, we could not differentiate between flexible and semi-rigid ureteroscopies because the records did not mention the type of ureteroscopy used. Third, because this study design was retrospective, selection bias may have been unavoidable.

## Conclusions

Unlike many literature reports, the observed pattern shows that the management during the lockdown period did not differ from the original recommendations and only three individuals (2.2%) did not receive the suggested management. We hypothesise that this is due to most patients having stone sizes between 5 and 10 mm and consequently managed by METs. Hence why, larger studies need to be conducted to provide concrete evidence.
